# Microtomography of the Baltic amber tick *Ixodes succineus* reveals affinities with the modern Asian disease vector *Ixodes ovatus*

**DOI:** 10.1186/s12862-016-0777-y

**Published:** 2016-10-10

**Authors:** Jason A. Dunlop, Dmitry A. Apanaskevich, Jens Lehmann, René Hoffmann, Florian Fusseis, Moritz Ehlke, Stefan Zachow, Xianghui Xiao

**Affiliations:** 1 Museum für Naturkunde, Leibniz Institute for Research on Evolution and Biodiversity, Invalidenstrasse 43, D-10115 Berlin, Germany; 2 United States National Tick Collection, The James H. Oliver, Jr. Institute for Coastal Plain Science, Georgia Southern University, Statesboro, GA 30460-8056 USA; 3 Faculty of Geosciences, University of Bremen, D-28357 Bremen, Germany; 4 Department of Earth Sciences, Ruhr-Universität Bochum, Institute of Geology, Mineralogy, and Geophysics, D-44801 Bochum, Germany; 5 School of Geosciences, The University of Edinburgh, Grant Institute, King’s Buildings, West Mains Road, Edinburgh, EH9 3JW UK; 6 Medical Planning Group, Zuse Institute Berlin, Department Visualization and Data Analysis, D-14195 Berlin, Germany; 7 Advanced Photon Source, Argonne National Laboratory, 9700 S. Cass Ave, Lemont, IL 60439 USA

**Keywords:** Arachnida, Parasitiformes, Ixodida, Parasite, Vector, Eocene, Baltic amber

## Abstract

**Background:**

Fossil ticks are extremely rare and *Ixodes succineus* Weidner, 1964 from Eocene (ca. 44–49 Ma) Baltic amber is one of the oldest examples of a living hard tick genus (Ixodida: Ixodidae). Previous work suggested it was most closely related to the modern and widespread European sheep tick *Ixodes ricinus* (Linneaus, 1758).

**Results:**

Restudy using phase contrast synchrotron x-ray tomography yielded images of exceptional quality. These confirm the fossil’s referral to *Ixodes* Latreille, 1795, but the characters resolved here suggest instead affinities with the Asian subgenus *Partipalpiger* Hoogstraal et al., 1973 and its single living (and medically significant) species *Ixodes ovatus* Neumann, 1899. We redescribe the amber fossil here as *Ixodes* (*Partipalpiger*) *succineus*.

**Conclusions:**

Our data suggest that *Ixodes ricinus* is unlikely to be directly derived from Weidner’s amber species, but instead reveals that the *Partipalpiger* lineage was originally more widely distributed across the northern hemisphere. The closeness of *Ixodes* (*P.*) *succineus* to a living vector of a wide range of pathogens offers the potential to correlate its spatial and temporal position (northern Europe, nearly 50 million years ago) with the estimated origination dates of various tick-borne diseases.

**Electronic supplementary material:**

The online version of this article (doi:10.1186/s12862-016-0777-y) contains supplementary material, which is available to authorized users.

## Background

Ticks (Acari: Ixodida) are iconic arachnids, being obligate ectoparasites of vertebrates and often of considerable economic significance as disease vectors affecting humans and/or domestic animals. For a recent summary of their biology see Sonenshine & Roe [1]. Almost 900 living species of ticks in three families (Nutalliellidae, Argasidae and Ixodidae) are currently recognized [2] and their origins and evolution remain a topic of debate [3–5]. Key questions include whether ticks share a common ancestor with mesostigmatid mites – or a lineage thereof [6] – or alternatively with the large and rare holothyrid mites [7]. Second, when did ticks originate, given that modern taxa require terrestrial vertebrate hosts, and on which palaeocontinent did they first appear? Recent opinions include an inferred Gondwanan (specifically South African) origin [8], in the Permian, although the same authors subsequently preferred an East African origin in the Carboniferous [9]. Other data suggest Australia, perhaps as far back as the Devonian [10]. Finally what, therefore, were their initial hosts? The monotypic and probably basal family Nutalliellidae has been reported as feeding on lizards [8], but was later shown to be a more generalist parasite [11] with, for example, subadults found on mammals like elephant shrews [12]. Note that tortoises, mammal-like reptiles, birds, and perhaps even feathered dinosaurs, would have been possible hosts for at least the Mesozoic species.

Palaeontology has the potential to contribute to this debate by recording when and where particular lineages (families, genera) were present. The oldest acariform mites are Devonian, but the oldest parasitiform mites – the clade which includes the ticks – have only been recorded since the Cretaceous. Fossil ticks are extremely rare. The oldest are two hard tick species (Ixodidae) from the Cretaceous (ca. 99 Ma) Burmese amber from Myanmar [13, 14]. Both were assigned to extinct genera; albeit not by recognized tick specialists. Burmese amber also includes a report of the living genus *Amblyomma* (Klompen in [15]), but was not described at species level. Like this find, all other fossil ticks have been assigned to extant genera, implicit of a strong degree of morphological stasis. The next oldest record is a soft tick (Argasidae: *Carios*) from Cretaceous (ca. 92 Ma) New Jersey amber [16]. This is followed by an *Ixodes* from Eocene (ca. 44–49 Ma) Baltic amber [17], which forms the focus of the present study, as well as a record of *Hyalomma* from Baltic amber [5], albeit without formal description. A putative *Ixodes* from the contemporary Green River Formation of Wyoming, USA is a *nomen dubium* [18]. Miocene (ca. 16 Ma) Dominican Republic amber has yielded an extinct species of the soft tick *Ornithodoros* [19], plus two hard ticks effectively indistinguishable from living Neotropical *Ambylomma* species [20, 21]. The youngest records are subfossils (<1 Ma) and include a *Dermacentor* from the ear of a fossilized rhinoceros from Poland (Kulczyński in [22]) and an *Ixodes* in an ancient owl pellet from Argentina [23]. In both cases the specimens could be comfortably assigned to living species.

**Fig. 1:**
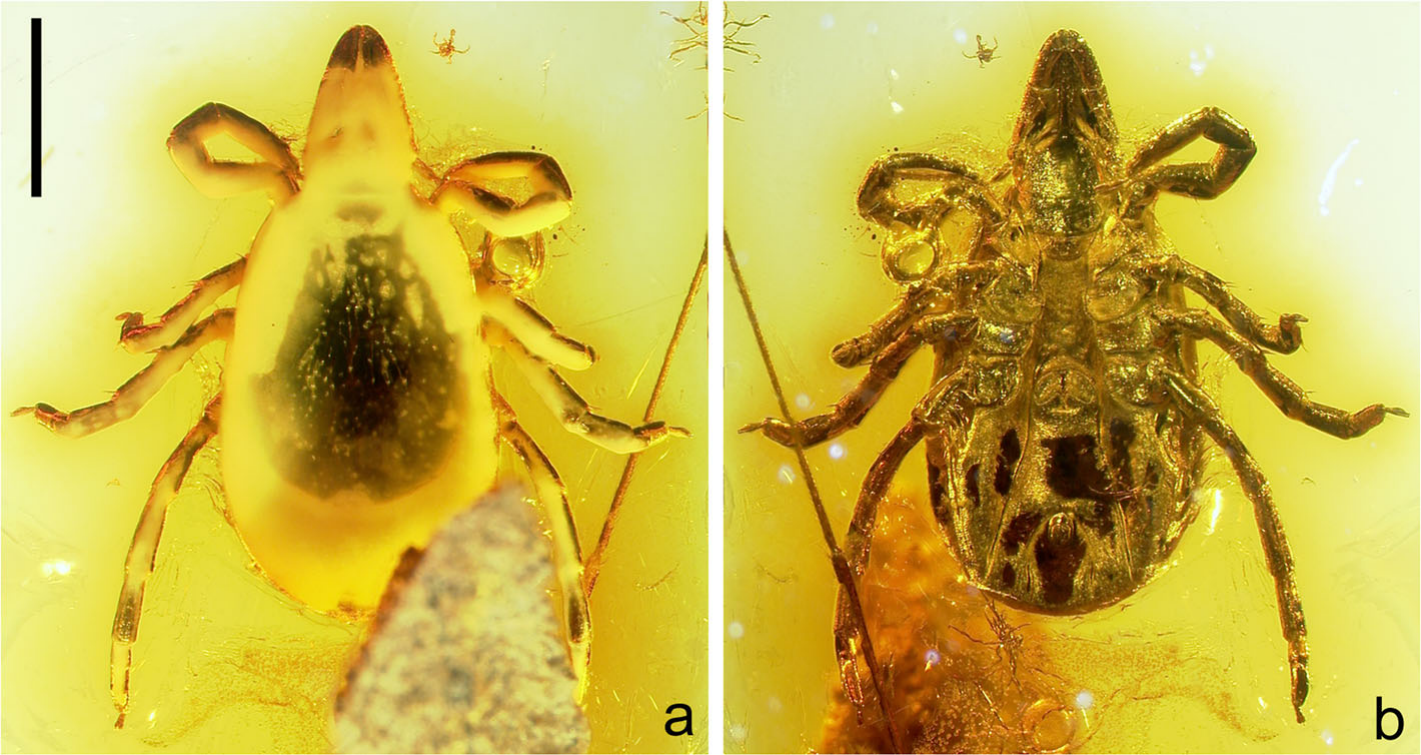
*Ixodes* (*Partipalpiger*) *succineus* Weidner, 1964. A fossil tick in Eocene (44–49 Myr) Baltic amber; holotype and only known specimen GSUB I21. **a** Light microscopy image in dorsal view; note the cloudy precipitate occluding much of the dorsal surface. **b** The same in ventral view. Scale bar 1.0 mm

*Ixodes succineus* Weidner, 1964 from Baltic amber (Fig. 1) is thus of particular significance as one of the oldest putative records of a living hard tick genus. We should also stress that numerous *Ixodes* species act as disease vectors today, thus understanding the origins of the genus is of wider parasitological interest. Including the amber fossil, Guglielmone et al. [24] recognized 244 valid *Ixodes* species and noted [see also 2] the, to some extent controversial [25], attempts to split the genus into subtaxa. The monophyly of *Ixodes* is by no means certain and a broad division into an Australasian and a non-Australasian clade has been proposed [26]. So where does the amber fossil fit into this scheme? Baltic amber is obviously geographically from northern Europe, but has been shown to occasionally pick up Asian or even Gondwanan faunal elements. Yet both the original description of *I. succineus* [17] and subsequent commentaries [27] remarked on similarities between the amber fossil and the common, Recent, largely European species *Ixodes ricinus* Linneaus, 1758; often known as the sheep, deer or castor bean tick. *Ixodes succineus* has been mentioned in tick catalogues [25] and refigured in popular works on amber [28–30], but a modern restudy of this important inclusion is lacking.

**Fig. 2:**
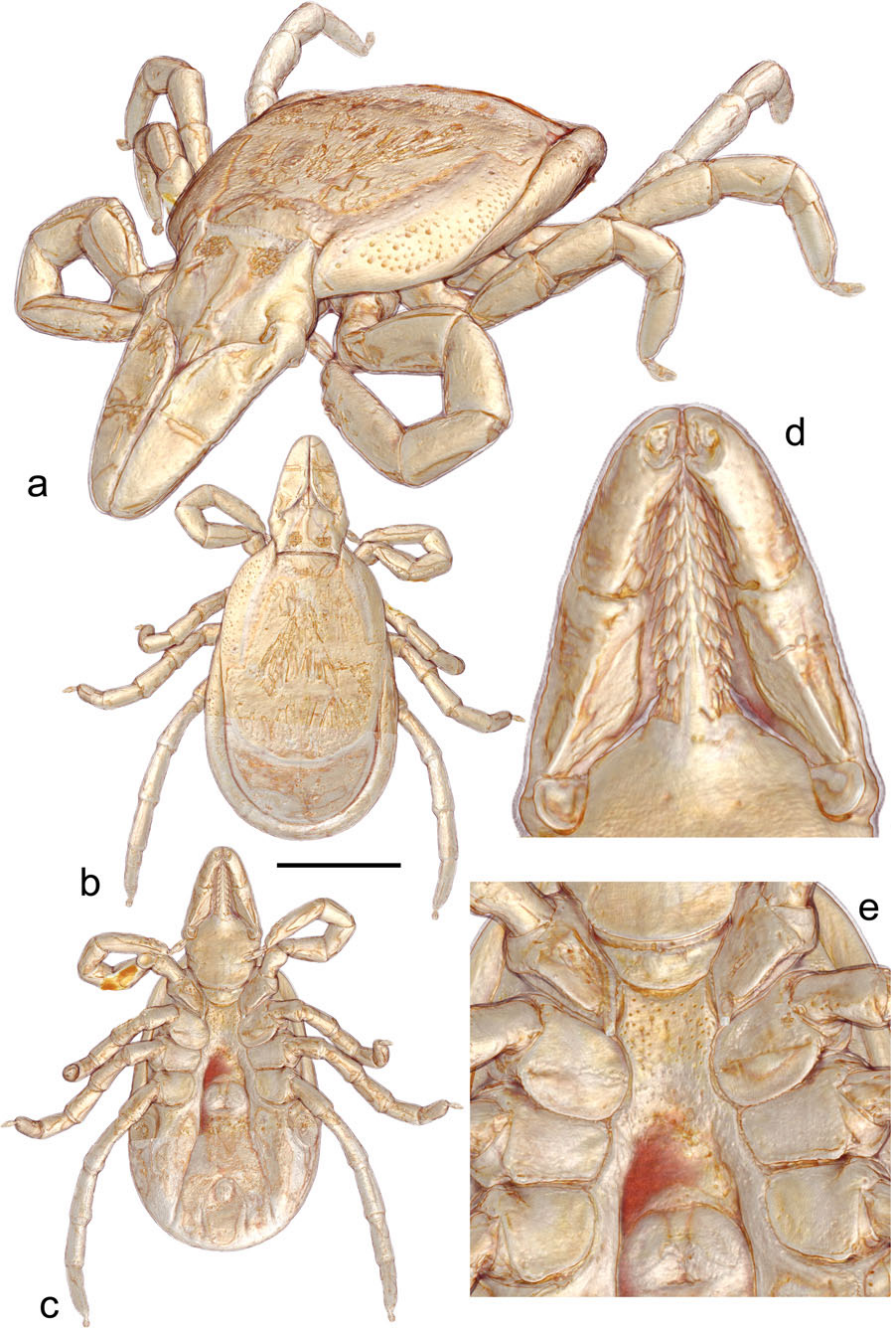
Tomographic renderings of *Ixodes* (*P.*) *succineus*. Numerous characters of taxonomic significance are now clearly revealed. **a** Oblique view. **b** Dorsal view. **c** Ventral view. **d** Details of gnathosoma. **e** Details of coxal region. Scale bar (for b and c) 1.0 mm

Fossils can be useful in constraining times of cladogenesis by acting as calibration points for molecular clocks [31, 32]. However, this requires accurate taxonomic placement, which in turn is facilitated by techniques which maximize the amount of morphological data recovered and allow the fossils to be placed using the same character sets as those applied to living genera and species. In recent years various forms of computed tomography (CT) have proved particularly well suited for imaging inclusions in amber, and have been successfully applied in acarology to both oribatid [33] and astigmatid mites [34]. Here, we offer the first CT study of a tick in amber. As well as producing extremely high-quality images of the inclusion (Fig. 2), our principal aim was to assess the fossil’s affinities – specifically is it an *Ixodes* and if so how close is it to *Ixodes ricinus* or to other living species? As noted above, *Ixodes* ticks often transmit diseases. Confirming the identity of the fossil would also allow us to compare this Eocene record with estimates of origination dates for pathogens typically carried by closely-related living tick species.

## Methods

### Material

The holotype and only specimen of *Ixodes succineus* derives from the legacy of a Baltic amber collection owned by the former physiology professor Otto Weiss (1871–1943) in Königsberg (now the Kaliningrad Oblast, Russia). It was purchased by the Überseemuseum Bremen, Germany in the early 1960s [17]. The geoscientific portion of this museum was transferred to the University of Bremen in 1994, thus the specimen is now held in the ‘Geowissenschaftliche Sammlung’ of the university under the inventory number GSUB I21. Baltic amber is conventionally dated to an Eocene (Lutetian) age of ca. 44–49 Ma and is thought to have been deposited in a warm forest environment [29]. The fossil was also examined under a stereomicroscope and compared both to Recent species held in the United States National Tick Collection and the literature.

### Tomography

Microtomographic data were collected at the bending magnet beam line 2-BM at the Advanced Photon Source, Argonne National Laboratory, USA. The amber specimen was scanned with 27.2 keV x-ray and imaged with a lens-coupled x-ray microscope. The microscope is composed of a Coolsnap K4 2048 × 2048 CCD camera, a 100um LuAG:Ce scintillator, and a Zeiss 5× objective lens. The scintillator converts the amber x-ray shadow image into an optical image, and the objective lens projects the image onto the camera. The microscope has a 2.96 μm pixel size. The exposure time was 200 ms. images were collected in transmission mode by a CCD camera behind the sample in the hutch configuration. The sample-detector distance was set to 300 mm to optimize the phase contrast which was needed to illustrate internal details of the poorly absorbing specimen; this distance being based on the evolution of the contrast in the reconstructed images. 1440 projections were acquired while the sample was rotated over 180° in steps of 0.125°. A microtomographic data set with a size of 2048x2048x1949 voxels (voxel size 2.96 μm) was reconstructed using a phase retrieval algorithm [35]. Digital visualization was achieved using ZIBAmira (https://amira.zib.de/). Synchrotron x-ray tomography techniques are known to run the risk of darkening the amber matrix surrounding the specimen, however no noticeable darkening was observed using the parameters listed above.

## Results

### Systematic palaeontology

Suborder IXODIDA Leach, 1815

Family IXODIDAE Leach, 1815

Genus *Ixodes* Latreille, 1795

*Ixodes (Partipalpiger) succineus* Weidner, 1964

### Material

Geowissenschaftliche Sammlung Universität Bremen (GSUB) I21, holotype (Figs. 1 and 2).

### Horizon and locality

Baltic amber, probably from the Kaliningrad region, Eocene (Lutetian).

### Emended diagnosis

Extinct member of the subgenus *Partipalpiger* Hoogstraal, Clifford, Saito & Keirans, 1973, which can be distinguished from the single living species by a 3/3 dental formula on almost the entire hypostome (2/2 in *Ixodes ovatus* Neumann, 1899) and a moderately long posteromedian spur on coxae I (short in *Ixodes ovatus*).

### Description

**Fig. 3:**
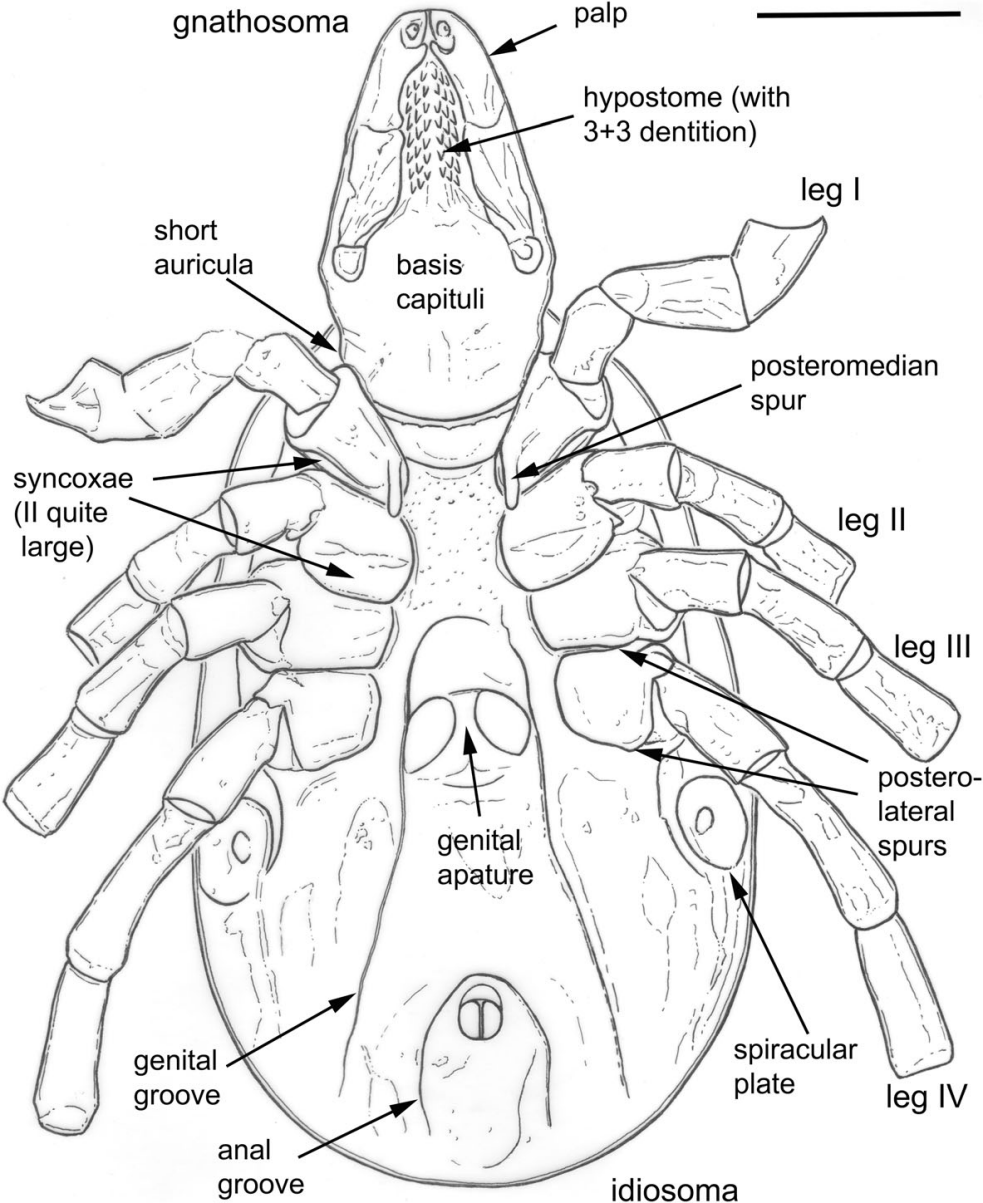
Interpretative drawing of *Ixodes* (*P*.) *succineus* in ventral view, highlighting taxonomically significant characters (see text for details). Scale bar 0.5 mm

Female tick. Idiosoma (Figs. 2 and 3): suboval, widest at level of coxae IV; length from scapular apices to posterior body margin 0.86 mm, breadth 0.56 mm, 1.54 times as long as broad. Scutum (Fig. 2b): Elongate, outline broadly rounded, length 0.64 mm, width 0.47 mm, 1.36 times as long as broad. Lateral carinae distinct, divergent, not reaching posterior margin; cervical grooves indistinct, shallow. Genital aperture medial to posterior margin of coxae IV; genital apron lacking. Genital groove well-developed. Anal groove circular with open posterior margin. Spiracular plates (Fig. 2b): sub-circular; diameter in anteroposterior plane slightly greater than that in dorsoventral plane; length 0.10 mm, width 0.09 mm, 1.11 times as long as broad.

Gnathosoma (Fig. 2d): length from palpal apices to cornual apices dorsally 0.34 mm, width at apices of lateral projections 0.23 mm, 1.48 times as long as broad. Basis capituli (Fig. 2b): dorsally subtriangular; posterior margin nearly straight; cornua small. Basis capituli ventrally pentagonal; with posterior margin convex; auriculae absent. Palpi (Fig. 2d): elongate, narrow; length (I–III segments) 0.26 mm, width 0.08 mm, 3.25 times as long as broad, length of segments in descending order: 2, 3, 1, 4; segment I well developed without spurs; segment II narrow proximally and gradually widening to mid-length and nearly parallel sided from mid-length to distal end; segment III laterally straight and medially converging to bluntly rounded apex. Hypostome (Fig. 2d): tapering with sharply pointed apex; arising from a medial anterior extension of basis; length 0.17 mm, width 0.05 mm, 3.4 times as long as broad; widest nearly at midlength; dental formula 3/3 throughout hypostomal length; denticles sharply pointed.

Legs: moderately long, slender. Coxae (Fig. 2e): coxae I with moderately long triangular posteromedian spur with sharply pointed apex, posterolateral spur lacking; coxae II without spurs; coxae III and IV with short triangular spur with blunt apex; coxae I–II with syncoxae; syncoxae on coxae II large, occupying posterior half of coxae. Tarsi (Fig. 2a): tarsi I–IV not humped at distal end; tarsus I length 0.22 mm; tarsus IV length 0.19 mm. Male, nymph and larva unknown.

### Remarks

GSUB I21 can be confirmed here both as a female fossil tick, and as the oldest unequivocal record of *Ixodes*. Based on the presence of syncoxae on coxae I and II (Figs. 2 and 3), the female of *I. succineus* most closely resembles members of the subgenera *Afrixodes* Morel, 1966, *Exopalpiger* Schulze, 1935 (including *Endopalpiger* Schulze, 1935), *Ixodes* s. str. and *Partipalpiger*. Species belonging to the *Afrixodes* subgenus – mostly distributed in the Afrotropical region and with a few species present in the Oriental region – have syncoxae present on coxae I–III and a genital apron. By contrast, the female of *I. succineus* has no syncoxae on coxae III and we were unable to find any trace of a genital apron. Most species of the subgenus *Exopalpiger*, mostly distributed in Australasian and Neotropical regions with a few species present in the Palaearctic and Afrotropics, also have coxae III and IV with syncoxae, no spurs on any coxae and palpal segment I with large projections. The female of *I. succineus* has syncoxae only on coxae I and II, a well-developed posteromedian spur on coxae I and posterolateral spurs on coxae III and IV, and a palpal segment I without large projections (Figs. 2 and 3).

Females of a number of species of the subgenus *Ixodes* s. str. also have syncoxae on coxae I and II only. These species can be found in the Palearctic, Oriental, Nearctic and Neotropical zoogeographic regions and probably do not form a natural group (see Discussion). We examined all valid species of *Ixodes* s. str. and were unable to find a modern species with a combination of characters exactly matching those in the amber fossil. In *Ixodes* s. str. species the syncoxae are considerably narrower and many of them have a posterolateral spur present on coxae II and/or larger and longer auriculae. By contrast, the female of *I. succineus* has very broad syncoxae on coxae II, no spurs on coxae II and very short and blunt auriculae (Figs. 2 and 3). It is the subgenus *Partipalpiger* – containing only *Ixodes ovatus* and distributed in the Palaearctic and Oriental regions from the Himalayas to Japan – which expresses a morphology most similar to the fossil in amber. Females of *I. ovatus* also have syncoxae only on coxae I and II and the syncoxae on coxae II are broad, occupying the posterior half of the coxae. Both the fossil and the extant species have very short, blunt auriculae with their apices oriented laterally. Nevertheless females of *Ixodes ovatus* can be easily distinguished from that of *I. succineus* by a 2/2 dental formula on almost the entire hypostome, as opposed to 3/3 in *I. succineus,* and a short posteromedian spur on coxae I, which is moderately long in *I. succineus* (Figs. 2 and 3).

## Discussion

### Affinities

Weidner’s original suggestion [17] that *Ixodes succineus* is closest to *Ixodes ricinus* could not be confirmed. It seems unlikely that this common, Recent, European species is derived directly from the lineage containing the Baltic amber inclusion. We see no obvious morphological continuum from the fossil to the *Ixodes* species which inhabits the Baltic region today. The evolutionary origins of the modern sheep tick must be sought elsewhere, and in any case these ectoparasites must surely have immigrated into north–central Europe after the last glaciation. Among recent molecular phylogenies of *Ixodes* [26, 36–38] it is worth noting the proposal that the genus is not monophyletic [26], and that ixodid ticks in general originated either in Africa or Australia. Our data confirms that *Ixodes* had reached Europe by at least 49 Ma. Furthermore, Xu et al. [38] challenged the monophyly of the *Ixodes ricinus* species complex and suggested that these ticks underwent – at some stage – a rapid radiation. Perhaps this was associated with the availability of their mammalian hosts. Further studies are undoubtedly needed to unravel the history of the modern European *Ixodes* tick fauna, and fossils could be integral to this process. As has been argued for insects [39], resolving phylogenies associated with rapid radiations in deep time can be problematic. Amber fossils like *Ixodes succineus* can take on increased importance for deciphering the phylogeny of rapidly-radiating groups, as they can reflect extinct lineages which sample different portions of the original radiation.

Our microtomography results suggest instead that *Ixodes succineus* from Baltic amber is most closely related to a modern Asian species, *Ixodes ovatus*, and can potentially be assigned to the same subgenus, *Partipalpiger*. This in itself is interesting and suggests that around 50 million years ago the *Partipalpiger* lineage (or at least closely-related forms) was more widespread across the northern hemisphere but became, at some stage, extinct in Europe. *Partipalpiger* has been resolved as the sister group of the subgenus *Ixodes* (s. str.) [37] and if this is correct the latter subgenus must also date back at least ca. 50 Ma. We may note here the proposal of Filippova [40] that *Ixodes* (s. str.) dates back to the Palaeogene, with an ecological niche in mesophilic and moderately hydrophilic Cenozoic forests. An open question is whether the loss of lineages, i.e., *Partipalpiger*, from the Eocene of northern Europe also occurred tens of millions of years ago, or perhaps as recently as the Pliocene–Quaternary Ice Ages which began about ca. 2.6 Ma. Baltic amber is thought to have concurred with a warm period – the Paleocene–Eocene Thermal Maximum and Early Eocene Climatic Optimum – and is known to host a range of taxa now restricted to other biogeographical areas. Larsson [41] reviewed Baltic amber fossils belonging to lineages which survive today either in southern Africa or in Asia. We propose that *Ixodes succineus* can be added to the latter group. Another example among parasitiform mites would be the opilioacarid genus *Paracarus* which has been recorded from Baltic amber [42], but is found today only in Kazakhstan.

### Correlations with pathogen origins?

In a wider context, Poinar [43] reviewed a number of insects in amber – particularly mosquitos, sand flies and assassin bugs – which today carry medically significant pathogens, and in which these exceptionally preserved inclusions even retained direct evidence for the protozoans responsible for infection. In a similar vein, Poinar [44] claimed to have observed rickettsial-like bacteria in one of the Burmese amber ticks, implying a minimum age of ca. 99 million years for this host–bacterial association. Whether these structures are genuine internal features or, e.g., an artefact of a reticulate surface ornament under particular conditions of illumination needs to be checked against the original material. This was, unfortunately, not available for loan. Potential pathogens cannot be resolved in our Baltic amber tick, but like a number of *Ixodes* species, its closest living relative *I. ovatus* is medically significant. For a detailed account of its biology see Hoogstraal et al. [45]. *Ixodes ovatus* has been recorded as a vector for the *Borellia* spirochetes responsible for Lyme borreliosis (LB) [46], for the *Babesia* protozoans responsible for babesiosis [47, 48], the bacteria responsible for anaplasmosis, ehrlichiosis and rikettsia-related infections [49, 50], and for the tick-borne encephalitis virus (TBEV) [51].

It is thus interesting to speculate whether the closely-related amber species was also a significant (mammalian) disease vector? Mammals, of course, underwent an adaptive radiation in the aftermath of the end-Cretaceous mass extinction. De la Fuente et al. [52] reviewed the role of (living) ticks as vectors, and our present revision of *Ixodes* (*P*.) *succineus* places a potential host in the Eocene of Europe. It may be instructive to compare this date to estimates of origination for the protozoa, bacteria or viruses which are intimately associated either with *Ixodes,* or with hard ticks in general. For example, Florin-Christensen & Schnittiger [31] discussed the evolutionary history of piroplasmids (including *Babesia*) and ticks. Molecular clock data suggests that piroplasmids in general originated ca. 57 Ma – i.e., just prior to the age of our fossil – but that the radiation into subgroups, including the babesids, is somewhat younger: ca. 25 Ma. Taking the oldest Mesozoic ticks as a starting point, these authors suggested that piroplasmids presumably entered a tick host at some point between the Cretaceous and the Oligocene.

Lyme disease is strongly associated with *Ixodes* species today, although it remains to be resolved whether the spirochetes entered ticks only once, or on multiple occasions. As reviewed by Fukunaga et al. [37] the phylogeny of the *Borellia* pathogen responsible is quite concordant with the phylogeny of the *Ixodes* ticks which host them: for example *I. ovatus* today hosts *B. japonica*. When the ticks, and their pathogens, radiated is another question. Margos et al. [53] noted that a molecular clock for the evolution of the housekeeping genes of the causative agent in humans, *B. burgdorferi* – which is strongly associated with the *I. ricinus* complex – has not yet been established. Nevertheless, these authors suggested that its origins were in Europe and that various lineages of this pathogen may have separated a few (unspecified) millions of years ago. The TBEV virus may also be a fairly recent acquisition. It has been hypothesised that these flaviviruses crossed over from argasid ticks on seabirds into ixodid ticks in modern Eurasian forests, or that its emergence may be associated with climate change at the end of the last glaciation [54].

Further data is available for bacterial infections [55, 56] and both studies inferred a Jurassic–Cretaceous split into *Rickettsia* bacteria associated with protozoans and a lineage more typical for hematophagous arthropods. This would be consistent with Poinar’s Burmese amber tick record (ca. 99 Ma) and his putative *Rickettsia*-like inclusions. Interestingly, both these *Rickettsia* molecular studies also reported a rapid radiation of this bacterial genus among blood-feeding taxa going back to ca. 50–65 Ma; more or less contemporary with the Baltic amber fossil but also, of course, with a radiation of possible mammalian hosts. Similarly, two medically significant strains of *Anaplasma* (*A. marginale* and *A. phagocytophilum*) were dated [57] as having split ca. 43–78 Ma.

## Conclusions

We cannot prove that *Ixodes succineus* carried bacteria, or other pathogens, but our study does confirm that at least one anatomically modern species of *Ixodes* was already present during the Eocene. It is closely related to a modern disease vector and can be placed in the same subgenus (*Partipalpiger*) as the Asian tick *Ixodes ovatus*. Thus the amber fossil may have acted as a vector too. Molecular estimates of origination dates suggest that at least piroplasmids and the *Rickettsia* and *Anaplasma* bacteria should have been present around 50 million years ago as well. This begs a further question whether evolution and radiation events among the pathogens are more closely tied to the ticks – as one of their carriers – or to the mammals as their principal hosts and reservoirs.
